# When does accounting for gene–environment interactions improve complex trait prediction? A case study with *Drosophila* lifespan

**DOI:** 10.1093/g3journal/jkaf304

**Published:** 2025-12-16

**Authors:** Fabio Morgante, Francesco Tiezzi

**Affiliations:** Institute for Human Genetics, Clemson University, Greenwood, SC 29646, United States; Department of Genetics and Biochemistry, Clemson University, Clemson, SC 29634, United States; Institute for Human Genetics, Clemson University, Greenwood, SC 29646, United States; Department of Agriculture, Food, Environment and Forestry (DAGRI), University of Florence, Florence 50144, Italy

**Keywords:** G×E, polygenic scores, genomic prediction

## Abstract

Gene–environment interactions (G×E) have been shown to explain a non-negligible proportion of variance for a plethora of complex traits in different species, including livestock, plants, and humans. While several studies have shown that including G×E can improve prediction accuracy in agricultural species, no increase in accuracy has been observed in human studies. In this work, we sought to investigate the scenarios in which accounting for G×E is expected to improve prediction accuracy. Model organisms are useful for studying G×E, since environments can be defined precisely, and genotypes can be replicated across environments, which are ideal conditions to minimize confounding in G×E analyses. Thus, we used data from an experiment in *Drosophila melanogaster*, where researchers measured lifespan in different environments for unrelated inbred lines (i.e. genotypes). We used three different cross-validation (CV) scenarios that mimic different relationships between reference and test populations, and fitted a few statistical models with and without including G×E. The results showed that G×E explained 8% of lifespan variance. Despite that, models accounting for G×E improved prediction accuracy only in CV scenarios where the same genotypes are observed in both the reference and test populations. While these scenarios are common in agriculture, where individuals of the same family or variety appear in both populations, they are not commonly encountered in human studies, where individuals are unrelated. Thus, our work shows in which prediction scenarios we can expect improvements by accounting for G×E, and may provide a potential reason (among others) for results of human studies.

## Introduction

Estimating the importance of gene–environment interactions (G×E) on complex traits and accounting for it in phenotype prediction is one of the most difficult challenges faced by plant and animal breeders (Bandeira e Sousa et al. 2017). Recently, this topic has also become of interest in human genetics (Kerin and Marchini 2020; Zhou and Lee 2021; Durvasula and Price 2025; Goda et al. 2025; Miao et al. 2025; Tiezzi et al. 2025).

In plant breeding, where G×E has the largest impact and has been studied most extensively, the estimation of such interaction largely relies on multi-environment trials (Xavier et al. 2025). Here, some varieties of a given species are planted in multiple locations, managed under different agronomic techniques and exposed to different pedo-climatological conditions. The estimates of G×E will be non-null when the performance of a given genotype varies across the different locations (Xavier et al. 2025). This implies that the genotype is sensitive to environmental conditions in its expression. G×E can result in different phenotypic variance across genotypes, depending on the environmental conditions in which the genotypes are reared, while the rank of the genotypes remains the same. However, G×E can also affect the rank of the genotypes across environments (Van Eeuwijk et al. 2016). Non-linearity of the response of the genotypes with respect to the environmental gradient adds to the complexity of G×E (Heslot et al. 2014).

Plant breeders leverage this interaction for developing cultivars that thrive in specific conditions or, perhaps, can perform well in multiple conditions (Elias et al. 2016; Van Eeuwijk et al. 2016). Similarly, animal breeders aim to estimate how the same or similar genotypes can acclimate to different conditions, by using data from related individuals (e.g. paternal half-sibs) that are raised in different environments (Bryant et al. 2005; Hansen 2020). This is done mostly to breed for animals that can be tolerant to stress (e.g. heat stress), but also to develop “the right genotype for that environment”, like ruminants that can adapt well to grazing conditions (Sheahan et al. 2011).

Many statistical approaches for plant and animal breeders have been developed (Hu et al. 2025). These approaches can be summarized as: (i) multiple trait models (MTMs) that consider the trait of interest as a series of correlated traits, each defined by the environment under which the trait is manifested; (ii) random regression models (RRM) that model the change in the performance across environments through the use of covariance functions, with conditions described by one or a few environmental covariates; and (iii) Reproducing Kernel Hilbert Space regression (RKHS) models that can handle a large number of environmental covariates in interaction with genotypes through the use of similarity matrices (i.e. kernels). All these models have been described in the literature, and the relationships between them can be demonstrated, at least in theory (Jarquín et al. 2014; Bandeira e Sousa et al. 2017; Martini et al. 2020; Hu et al. 2025). It should be noted that MTM is the least parsimonious in terms of parameters to estimate when the number of environments or environmental descriptors becomes large. On the other hand, RKHS is the most parsimonious, as it was developed specifically to handle a large number of environmental covariates (Jarquín et al. 2014). Appropriate modeling of G×E is pivotal in understanding the complexity of this phenomenon, and different strategies have been proposed to evaluate the different methods. These strategies include benchmarking the prediction methods for their ability to rank genotypes regardless of the environmental conditions, or within specific conditions (e.g. within-year-location correlation) (Lopez-Cruz et al. 2023).

Models that incorporate G×E have shown good promise in plant and animal breeding. For example, in plants, Jarquín et al. (2017) showed that G×E improved prediction accuracy when modeling the performance of wheat lines grown across dozens of locations, while Acosta-Pech et al. (2017) successfully modeled general and specific combining ability in interaction with the environment to predict the performance of maize hybrid lines. In addition, Cuevas et al. (2016) showed that G×E implemented in RKHS models produced advantages with non-null correlations between environments. In livestock, both Bohlouli et al. (2019) and Tiezzi et al. (2017) found a clear advantage in modeling G×E using climate data in dairy cattle and RRM or RKHS models, respectively. On the other hand, Bussiman et al. (2025) did not find a clear advantage in the inclusion of G×E in pigs.

Studies in plants also showed that the benefits of including G×E in prediction models are strongly dependent on the structure of and the relationship between the training and test populations (Crossa et al. 2014; Millet et al. 2019). For example, incorporating G×E showed the greatest advantage when predicting untested combinations of tested genotypes and tested environments (i.e. incomplete field trials). On the other hand, incorporating G×E did not show a marked improvement in prediction accuracy when predicting untested genotypes in tested environments (Burgueño et al. 2012) or tested genotypes in untested environments (Jarquín et al. 2017).

In human genetics, there are additional difficulties to study G×E because researchers cannot design optimal experiments and need to rely on large observational data such as biobanks (Bycroft et al. 2018). These data have a large amount of missing records, rely on noisy self-assessed information about the environment, lack replication of genotypes (i.e. individuals) across environments, and genotypes are non-randomized with respect to environments (Tiezzi et al. 2025). These problems make estimating the magnitude of G×E challenging. Despite that, non-negligible contribution of G×E to the phenotypic variance of several complex traits has been shown (Dahl et al. 2020; Kerin and Marchini 2020; Miao et al. 2025). However, accounting for G×E has generally not resulted in an increase in out-of-sample prediction accuracy (Zhou and Lee 2021; Goda et al. 2025; Tiezzi et al. 2025; Weine et al. 2025). These results disagree with those obtained in agricultural breeding.

To study in which scenarios accounting for G×E is expected to improve prediction accuracy and to investigate the contradictory results between humans and agricultural species, model organisms can provide valuable insights. As opposed to humans and, to some extent, agricultural species, experiments in model organisms can be carefully controlled. This results in precise environment definitions, accurate phenotypic measurements, and replication of genotypes across environments, which are ideal conditions for G×E analyses and minimize confounding. In this study, we used data from an experiment that measured lifespan for flies from the *Drosophila melanogaster* Genetic Reference Panel (DGRP) inbred lines raised in different environments (Huang et al. 2020). One peculiarity of these lines is that they are largely unrelated (Huang et al. 2014), which differs from populations used in similar studies performed in plants (Burgueño et al. 2012; Jarquín et al. 2017). Using these data and previously devised prediction scenarios, we sought to investigate the conditions in which accounting for G×E in different statistical models improves out-of-sample prediction accuracy.

## Materials and methods

### Data processing

We used phenotypic data from Huang et al. (2020). Lifespan (in days) was measured for 186 inbred lines of the DGRP for the two sexes at three different temperatures (18 ∘C, 25 ∘C, 28 ∘C). After removing lines that had missing values in at least a sex/temperature combination, we were left with n=176 lines, each measured in c=2 context variables (resulting in r=6 sex/temperature combinations), for a total of q=1,056 records. The genotype data were filtered to remove genetic variants with minor allele frequency (MAF) smaller than 0.05 and missing genotype rate greater than 0.2. These filters retained p=1,899,439 genetic variants.

### Statistical models

We analyzed the data using RKHS regression models described in Jarquín et al. (2014):

- G-BLUP. $y_{ij}=\mu +a_{i}+\varepsilon_{ij}$.
- E-BLUP. $y_{ij}=\mu +e_{\;j}+\varepsilon_{ij}$.
- GE-BLUP. $y_{ij}=\mu +a_{i}+e_{\;j}+\varepsilon_{ij}$.
- G×E-BLUP. $y_{ij}=\mu +a_{i}+e_{\;j}+ae_{ij}+\varepsilon_{ij}$.

Here, $y_{ij}$ is the phenotype of line *i* in environment *j*, *μ* is the intercept value, $a_{i}$ is the random additive genetic value of line *i* [$a∼N_{q}(0,ZGZ^{⊺}\sigma_{a}^{2})$, Z is a q×n incidence matrix with q=1,056 and n=176, G is a n×n genomic relationship matrix (GRM) computed as in VanRaden 2008], $e_{\;j}$ is the random environmental value of environment *j* [$e∼N_{q}(0,E\sigma_{e}^{2})$, E is a q×q matrix of similarity based on environmental variables, computed as $E\propto {XX}^{⊺}$, X is a q×c matrix of environmental measurements, with c=2], $ae_{ij}$ is the random gene–environment value of line *i* in environment *j* [$ae∼N_{q}(0,ZGZ^{⊺}\circ E\sigma_{ae}^{2})$], $\varepsilon_{ij}$ is the residual value for line *i* in environment *j* [$\varepsilon ∼N_{q}(0,I_{n}\sigma_{\varepsilon }^{2})$].

These models were fitted in a Restricted Maximum Likelihood (REML) framework, implemented in the sommer R package (Covarrubias-Pazaran 2016).

We also fit a MTM (Calus and Veerkamp 2011):

- mvG-BLUP.

$Y=1m^{⊺}+A+R$

Here, Y is an n×r matrix of phenotypic observations in the r=6 environments, **1** is an *n*-vector of ones, m is an *r*-vector of intercept values, A is an n×r matrix of additive genetic values [$A∼{MN}_{n\times r}(0,G,\Sigma_{A})$, $\Sigma_{A}$ is an r×r genetic covariance matrix], R is an n×r matrix of residual values [$R∼{MN}_{n\times r}(0,I_{n},\Sigma_{R})$, $\Sigma_{R}$ is an r×r diagonal residual covariance matrix].

This model was fitted in a Bayesian framework, implemented in the BGLR R package (Pérez and de Los Campos 2014 ). We ran the sampler for a total of 300,000 iterations, discarding the initial 200,000 iterations as burn-in, followed by thinning every 50 iterations.

We also fit a RRM (Schaeffer 2004):

- RRM. $y_{ij}=\sum_{t=0}^{T}\beta_{t}\phi_{t}(j)+\sum_{t=0}^{T}a_{it}\phi_{t}(j)+\varepsilon_{ij}$

Here, $y_{ij}$ is the phenotype for line *i* in environment *j*, $\phi_{t}(j)$ is the Legendre polynomial of order *t* for environment *j*, $\beta_{t}$ is the fixed effect of the *t*th-order Legendre polynomial, $a_{it}$ is the random additive genetic value of the *t*th-order Legendre polynomial for line *i* [$a∼N_{q}(0,G⊗\Sigma_{a})$, $\Sigma_{a}$ is a T×T genetic covariance matrix], $\varepsilon_{ij}$ is the residual value for line *i* in environment *j* [$\varepsilon ∼N_{q}(0,I_{n}⊗\Sigma_{\varepsilon })$, $\Sigma_{\varepsilon }$ is an r×r diagonal residual covariance matrix].

We used the mean phenotype across lines within each environment as the environmental value to compute $\phi_{t}(j)$. After some investigation with polynomials of different orders (results not shown), we chose polynomials of order 1 (i.e. T = 1) as they provided better performance than higher order ones. In fact, Supplementary Fig. S1 shows that a linear approximation may suffice for most lines. This model was fitted in a Restricted Maximum Likelihood (REML) framework, implemented in the sommer R package (Covarrubias-Pazaran 2016).

### Validation schemes

We implemented three cross-validation (CV) schemes, whereby part of the data (i.e. the training set) was used to train the models and the remaining part (i.e. the test set) was used to evaluate prediction accuracy. These schemes were used previously in plant studies (Burgueño et al. 2012; Jarquín et al. 2017).

**Fig. 1.**
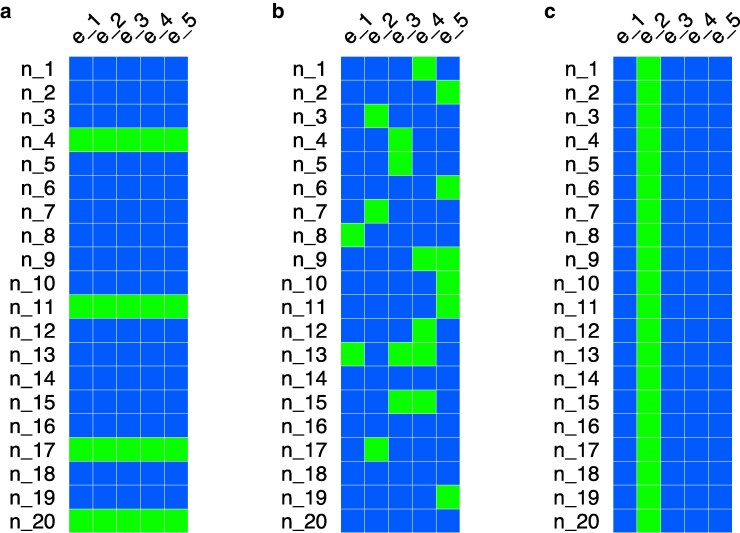
Cross-validation schemes used to evaluate the prediction accuracy of the different models. a) *Random Lines*. b) *Random Observations*. c) *New Environment*. The blue squares represent observations in the training set and the green squares represent observations in the test set.

1. *Random Lines* (similar to CV1 in Burgueño et al. 2012). We assigned 17% of the lines (for all the combinations of sex and temperature) to the test set, randomly (Fig. 1a). This procedure was repeated 6 times. The peculiarity of this scheme is that the lines in the test set are not represented in the training set. Thus, the training-test transfer of information happens mostly at the environmental level, when genotypes are unrelated.
2. *Random Observations* (similar to CV2 in Burgueño et al. 2012). We assigned 17% of the observations (i.e. combinations of line, sex, and temperature) to the test set, randomly (Fig. 1b). This procedure was repeated 6 times. The peculiarity of this scenario is that all the lines, sexes, and temperatures are represented in the training set. Thus, the training-test transfer of information happens at both the genetic and environmental level.
3. *New Environment* (similar to CV0 in Jarquín et al. 2017). We assigned all the observations in a specific combination of sex and temperature to the test set (∼17% of the data) (Fig. 1c). This procedure was repeated 6 times. The peculiarity of this scenario is that a sex/temperature combination (i.e. an environment) is never seen in the training set. Thus, while technically the training-test transfer of information happens at both the genetic and environmental level, for the latter, it is hampered by not observing the actual environment in which we are trying to predict.

We computed prediction accuracy as $R^{2}$ from the regression of the true phenotypes on the predicted phenotypes, averaged over the 6 replicates. For the *Random Lines* and *Random Observations* scenarios, we also computed $R^{2}$ within each environment and then computed the average across environments (Lopez-Cruz et al. 2023).

## Results and discussion

**Fig. 2.**
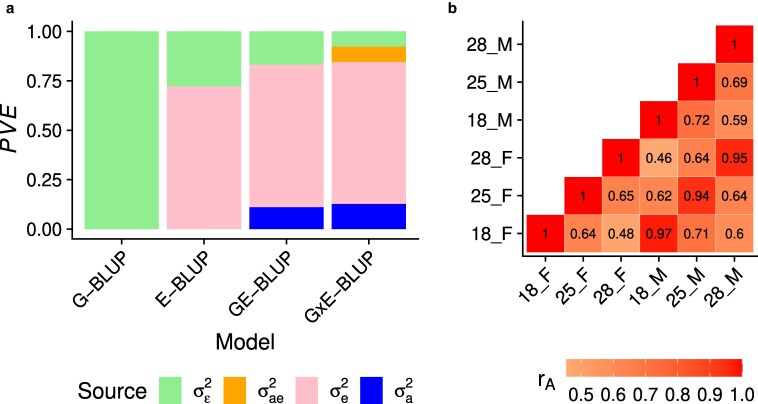
Variance partition and cross-environment genetic correlations of lifespan. a) Variance partition into genetic ($\sigma_{a}^{2}$), environmental ($\sigma_{e}^{2}$), gene–environment interaction ($\sigma_{ae}^{2}$), and residual ($\sigma_{\varepsilon }^{2}$) components using RKHS models. The y-axis shows proportion of variance explained (PVE). b) Cross-environment genetic correlations ($r_{A}$) estimated using MTM.

We first partitioned the phenotypic variance into sources of variation attributed to genetics, environment, and gene–environment interactions (Fig. 2a).

The results (Fig. 2a) show that the environment explained the largest amount of lifespan variance (∼72%) across all models that include that component. The genetic variance was the second largest contributor in GE-BLUP and G×E-BLUP, explaining ∼12% of the variance. However, genetic effects explained no variance in G-BLUP. We attributed this result to the fact that when the environment explains most of the variance and its effect is not modeled, the model struggles to find the (minor) genetic signal in the large amount of unexplained variance. But once the effect of the environment is included in GE-BLUP and G×E-BLUP, it is easier for genetic effects to explain some of the remaining variance (i.e. not accounted for by the environment). G×E explained ∼8% of the phenotypic variance. Importantly, when including G×E in the model, it explained variance that would otherwise be included in the residual, as implied by the similar proportion of variance explained (PVE) by genetic effects and environmental effects in GE-BLUP and G×E-BLUP. We also sought to confirm the presence of G×E in a complementary analysis, where we estimated cross-environment genetic correlations ($r_{A}$), treating lifespan in each of the six environments (i.e. sex/temperature combinations) as a different trait in mvG-BLUP (Calus and Veerkamp 2011). The results (Fig. 2b) show that the genetic correlations were different from unity for every pair of environments, especially across temperatures. The observation that genetic effects are different across environments agrees with the presence of G×E (Falconer and Mackay 1996).

**Fig. 3.**
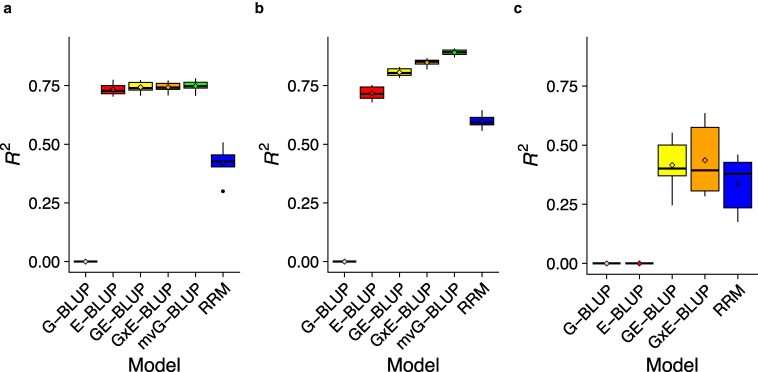
Prediction accuracy in the different cross-validation schemes. a) *Random Lines*. b) *Random Observations*. c) *New Environment*.

We then assessed the accuracy of the different models at predicting yet-to-be-observed phenotypes using three CV schemes, illustrated in Fig. 1. The prediction results are shown in Fig. 3.

In the *Random Lines* scenario (Fig. 3a), G-BLUP performed poorly. This result is expected, since the lines assigned to the test were not present in the training set, DGRP lines are largely unrelated, and G-BLUP was shown to explain no variance in the whole dataset. On the other hand, E-BLUP had high prediction accuracy ($R^{2}\approx 0.72$). Here, sex and temperature were shown to explain ∼72% of the variance and all the sex/temperature combinations were observed in the training set, allowing to obtain precise estimates of their effects. The prediction accuracy provided by GE-BLUP and G×E-BLUP was similar to (although slightly higher than) that of E-BLUP, showing that the inclusion of genetic effects and gene–environment interaction effects did not result in a substantial improvement of the predictions in this scenario. mvG-BLUP provided similar accuracy to E-BLUP, GE-BLUP, and G×E-BLUP. This result stems from the fact that mvG-BLUP fits lifespan in each environment as a different trait and thus estimates environment-specific intercepts, genetic and residual variances as well as genetic and residual correlations across environments. The difference in phenotype due to the different environmental conditions is accounted for by the intercepts in mvG-BLUP. The genomic heritability ranged ∼72−80% across environments, showing that genetic effects could explain a large proportion of within-environment phenotypic variance. mvG-BLUP also leveraged the medium-high cross-environment genetic correlations to achieve good prediction accuracy. On the other hand, RRM had a substantially lower accuracy compared to the other environment-aware models. These results are in partial disagreement with other studies that showed RRM to be competitive with the other models (Sun et al. 2017). An explanation for this observation is that our data might be suboptimal for the application of RRM, since the environments appear to be better defined as discrete rather than stratified on a continuous scale. RRM might also be better suited for the analysis of a larger number of environments than in our study, which allows for a better estimation of trajectories (Jarquín et al. 2017). In fact, RRM were developed for modeling longitudinal data such as test day milk production in dairy cattle (Schaeffer 2004).

In the *Random Observations* scenario (Fig. 3b), prediction accuracies were generally higher than in the *Random Lines* scenario. This is expected since all the lines and environments were present in the training set—only specific line/environment combinations were not observed in the training set. Thus, *Random Observations* is a considerably less challenging scenario than *Random Lines* for prediction. Similar to the *Random Lines* scenario, G-BLUP achieved null accuracy, whereas E-BLUP performed well ($R^{2}\approx 0.72$). However, GE-BLUP improved accuracy substantially over E-BLUP, providing $R^{2}\approx 0.81$ in this scenario. This shows that once the large proportion of variance explained by the environment is accounted for, genetic effects can explain additional variance and improve prediction accuracy in this scenario. Accounting for G×E in G×E-BLUP increased accuracy further ($R^{2}\approx 0.85$), showing that this full model can achieve high accuracy at predicting specific genotype/environment combinations by borrowing information across lines and across environments. The best performing model was mvG-BLUP, which achieved $R^{2}\approx 0.89$. This is a remarkable result since $R^{2}$ from this model closely approached the PVE of the full model (i.e. G×E-BLUP) in the whole dataset (∼0.92). Thus, a model that explicitly accounts for the specificity of genetic effects across environments, while also leveraging their similarities, seems to be the best choice. However, this model has some limitations: (1) it requires discrete, well defined environments, with a low to moderate number of classes; (2) it conflates the contributions of genetics and G×E to the phenotypic value into a single term; (3) it does not allow for the prediction in unobserved environments, unless estimates of the genetic and residual (co)variances between observed and unobserved environments, and the phenotypic mean in the unobserved environments are available. Again, RRM had a much lower accuracy compared to the other environment-aware models in this scenario.

For the these two CV schemes, we also computed within-environment $R^{2}$. This metric is particularly relevant to plant studies, where environments are well defined. The results (Supplementary Fig. S2) show that prediction accuracy was much lower with this metric compared to its pooled-environments counterpart, especially in the *Random Lines* scenario. This result can be explained by the fact that within-environment $R^{2}$ is not influenced by the difference in phenotypic means between environments, and here the environment was the largest contributor to the phenotypic variance. The general patterns observed with pooled-environments $R^{2}$ (Fig. 3) were also observed with within-environment $R^{2}$. In the *Random Lines* scenario, accounting for G×E did not improve prediction accuracy. In the *Random Observations* scenario, accounting for G×E improved prediction accuracy when using mvG-BLUP. However, G×E-BLUP performed as well as GE-BLUP, which is in contrast with the results of the pooled-environments $R^{2}$.

The *New Environment* scenario (Fig. 3c) was the most challenging of the CV schemes, resulting in considerably lower prediction accuracies compared to the other scenarios. In fact, the environment in which we are trying to predict is not observed in the training set, and the environmental conditions explain the majority of the phenotypic variance. This is reflected in E-BLUP yielding null accuracy in this scenario. However, GE-BLUP achieved moderate accuracy ($R^{2}\approx 0.42$), presumably due to a better disentanglement of genetic and environmental effects when estimating parameters in the training set. G×E-BLUP provided slightly improved accuracy ($R^{2}\approx 0.44$) compared to GE-BLUP, confirming the utility of accounting for G×E when predicting in unobserved environments. In this scenario, mvG-BLUP could not be fitted because of reason (3) discussed in the previous paragraph. While RRM performed worse than GE-BLUP and G×E-BLUP on average, its accuracy ($R^{2}\approx 0.34$) was much closer to the accuracies of those models in this scenario. In the *New Environment* scenario, there were pronounced differences in prediction accuracy depending on which environment the predictions were made (Table 1). Overall, at least one of the models accounting for G×E (i.e. G×E-BLUP and RRM) performed as well as or better than GE-BLUP in every environment. As expected given the estimates of the cross-environment genetic correlations, differences in prediction accuracy were larger among temperatures than between sexes (within temperature). At 18 ∘C, G×E-BLUP greatly outperformed GE-BLUP. At 25 ∘C, G×E-BLUP and GE-BLUP yielded similar accuracy. RRM performed substantially worse than the other methods at both 18 ∘C and 25 ∘C. On the other hand, at 28 ∘C, RRM achieved higher accuracy than GE-BLUP and G×E-BLUP.

**Table 1.** Prediction accuracy ($R^{2}$) in each environment (Sex_Temperature) in the *New Environment* cross-validation scenario.

Env	G-BLUP	E-BLUP	GE-BLUP	G×E-BLUP	RRM
F_18 ∘C	0.00	0.00	0.55	0.63	0.37
M_18 ∘C	0.00	0.00	0.52	0.63	0.44
F_25 ∘C	0.00	0.00	0.37	0.37	0.17
M_25 ∘C	0.00	0.00	0.43	0.42	0.19
F_28 ∘C	0.00	0.00	0.25	0.29	0.46
M_28 ∘C	0.00	0.00	0.37	0.28	0.39

Overall, our results showed that accounting for G×E can be helpful for improving predictions. However, the presence and magnitude of the improvement depended on the CV scenario, as shown in previous studies (Burgueño et al. 2012; Jarquín et al. 2017). If the goal is to predict phenotypes for unknown individuals in known environments (our *Random Lines* scenario), accounting for G×E may not improve prediction accuracy. An explanation for this result is the lack of information sharing at the genetic level between the training set and the test set, when the individuals in the test set are largely unrelated to the individuals in the training set. In fact, Jarquín et al. (2017) found that a model with G×E outperformed the models without it in a similar CV scenario in wheat, which may be due to higher genetic similarity between training and test sets.

At the other end of the spectrum, if the goal is to predict phenotypes for a set of known individuals in a new environment (our *New Environment* scenario), accounting for G×E may improve prediction accuracy. A plausible explanation for this observation is that G×E-aware models can predict the adaptability of each genotype to several conditions. This scenario is particularly relevant for agricultural breeding, where breeders need to know how available breeds/cultivars would fare in a new environment (Reynolds et al. 2018; Tiezzi and Maltecca 2022), and how accurately models can predict such performance. However, the models’ predictive ability to new environments can depend on the environment itself in at least two ways. First, phenotypes could be easier to predict in certain environments, if these show stronger genetic correlations with the known environments (Burgueño et al. 2012) and the models can borrow information across environments (Crossa et al. 2014). This is also in agreement with Lopez-Cruz et al. (2023), who showed that the within-year-location accuracy had some spread, indicating that the predictive ability depended on the environmental conditions. Second, different G×E-aware methods may outperform others, depending on which environment we aim to predict in (as seen in Table 1).

Finally, if the goal is to predict unobserved phenotypes for a set of known individuals in known environments (our *Random Observations* scenario), that is the scenario where we can expect the largest increase in accuracy when accounting for G×E. This is in agreement with previous studies (Burgueño et al. 2012; Jarquín et al. 2017). In this scenario, a multivariate model treating phenotypes in different environments as different traits seemed to provide the highest accuracy. The *Random Observations* scenario is particularly relevant to precision medicine in humans, where there is interest in predicting medically relevant phenotypes (e.g. blood pressure) after a change in the environment (e.g. switching from a high fat diet to a low fat diet). However, this scenario cannot be evaluated with human data, as individuals are present in only one level of the environmental variable at any given time (e.g. a person either smokes or does not, but not both). This peculiarity might also contribute to the reasons why accounting for G×E—despite explaining non-negligible variance—has generally not resulted in improved prediction accuracy for human traits (Zhou and Lee 2021; Goda et al. 2025; Tiezzi et al. 2025), as well as some livestock species (Bussiman et al. 2025). In fact, assigning some individuals to the test set and trying to predict their phenotype in a known environment (as done in human studies) is equivalent to our *Random Lines*. In this scenario, we have shown that including G×E does not improve predictions, when training individuals and test individuals are largely unrelated as it is common in human studies.

However, it should be noted that human studies, which are observational in nature, have additional complications that could contribute to the lack of prediction accuracy improvement from accounting for G×E. First, environments are not clearly defined, which may result in environmental heterogeneity. Second, phenotypic and, especially, environmental measures are noisy. Thus, considering that interaction effects are usually smaller than main effects, there may be limited statistical power to estimate G×E effects precisely (which is necessary for accurate predictions) with current sample sizes of human studies. Third, non-random allocation of individuals (i.e. genotypes) to environments might result in reverse causality or genotype–environment correlation, which confound estimates of G×E (Tiezzi et al. 2025).

Our study has some important limitations. First, the DGRP has a small sample size, limiting the accuracy with which genetic effects can be estimated. Second, all the models used in this study do not perform variable selection, implying that all genetic variants, all environmental variables, and all the interactions between them have an effect on lifespan. Using methods that perform variable selection has the potential to increase prediction accuracy further. Third, our study focused on only one trait, as this was the only one available for a well designed and controlled experiment including unrelated genotypes, which allowed us to avoid confounding effects. Thus, our results will need to be confirmed on additional traits from equally well designed, but larger experiments.

## Supplementary Material

jkaf304_Supplementary_Data

## Data Availability

The genotype data are available from Huang et al. (2014). The phenotype data are available from Huang et al. (2020). Code used for the analyses is available at https://github.com/morgantelab/dgrp_lifespan_gxe. Supplemental material available at G3 online.
