# Supplementary material for: When does accounting for gene–environment interactions improve complex trait prediction? A case study with Drosophila lifespan
Source: G3 (Bethesda). 2025 Dec 16;16(2):jkaf304. doi: 10.1093/g3journal/jkaf304 (PMC12869066; doi:10.1093/g3journal/jkaf304)
Supplement: jkaf304_Supplementary_Data [file jkaf304_supplementary_data.pdf]

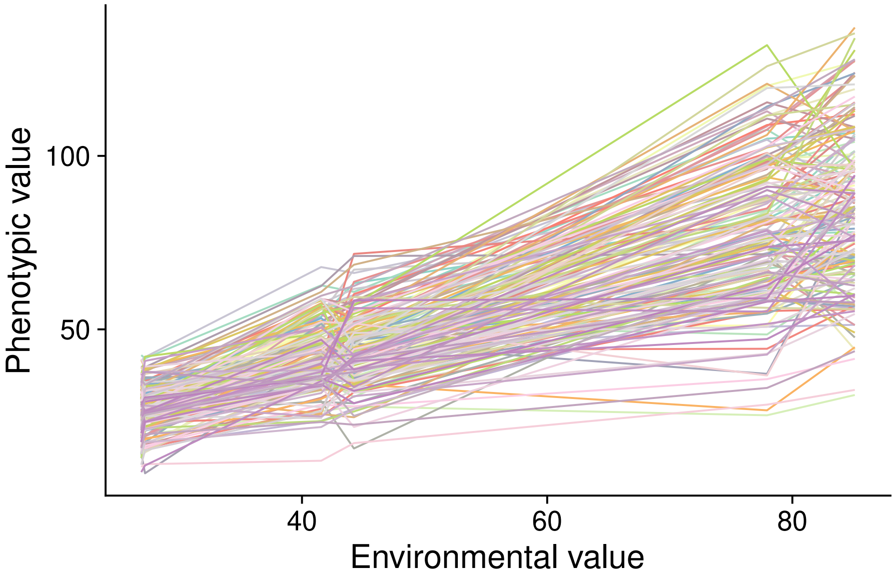

Figure S 1: Reaction norm for each line. The environmental value is computed as the phenotypic mean across lines within each environment.

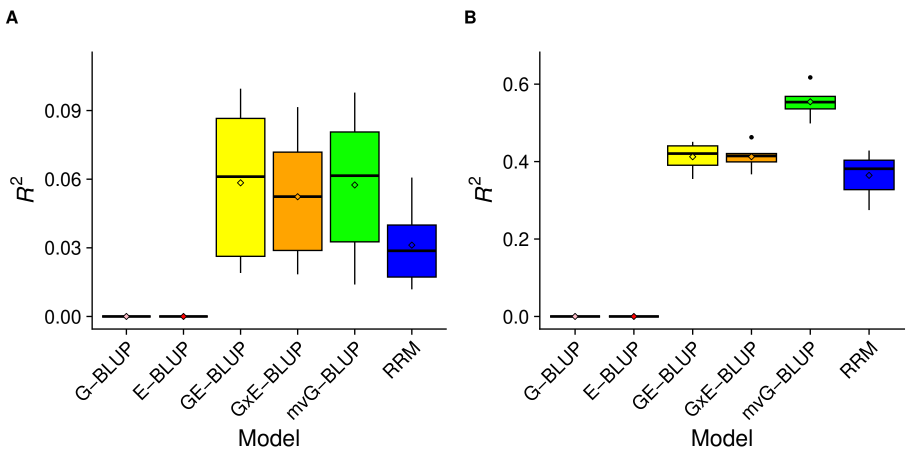

Figure S 2: Within-environment prediction accuracy in the different cross-validation schemes. A) *Random Lines*. B) *Random Observations*.
